# An online tool for mapping insecticide resistance in major *Anopheles* vectors of human malaria parasites and review of resistance status for the Afrotropical region

**DOI:** 10.1186/1756-3305-7-76

**Published:** 2014-02-21

**Authors:** Tessa B Knox, Elijah O Juma, Eric O Ochomo, Helen Pates Jamet, Laban Ndungo, Patrick Chege, Nabie M Bayoh, Raphael N’Guessan, Riann N Christian, Richard H Hunt, Maureen Coetzee

**Affiliations:** 1 Vestergaard Frandsen (Ltd.) East Africa, PO Box 66889–00800, Nairobi, Kenya; 2 Vestergaard Frandsen SA, Chemin de Messidor 5-7, CH– 1006 Lausanne, Switzerland; 3 KEMRI/CDC Research and Public Health Collaboration, P.O. Box 1578, Kisumu 40100, Kenya; 4 Department of Biomedical Sciences, Maseno University, Maseno, Kenya; 5 Esri Eastern Africa, P.O. Box 57783, Nairobi 00200, Kenya; 6 Disease Control Department, London School of Hygiene and Tropical Medicine, Keppel Street, London WC1E 7HT, UK; 7 Centre de Recherche Entomologique de Cotonou, Cotonou 06 BP 2604, Benin; 8 Wits Research Institute for Malaria, School of Pathology, Faculty of Health Sciences, University of the Witwatersrand, Johannesburg, South Africa; 9 Vector Control Reference Laboratory, National Institute for Communicable Diseases of the National Health Laboratory Service, Sandringham, Johannesburg, South Africa

**Keywords:** Africa, *Anopheles*, Insecticide resistance, IR Mapper, Mechanisms, Malaria, Target site

## Abstract

**Background:**

Malaria control programmes across Africa and beyond are facing increasing insecticide resistance in the major anopheline vectors. In order to preserve or prolong the effectiveness of the main malaria vector interventions, up-to-date and easily accessible insecticide resistance data that are interpretable at operationally-relevant scales are critical. Herein we introduce and demonstrate the usefulness of an online mapping tool, IR Mapper.

**Methods:**

A systematic search of published, peer-reviewed literature was performed and *Anopheles* insecticide susceptibility and resistance mechanisms data were extracted and added to a database after a two-level verification process. IR Mapper ( http://www.irmapper.com) was developed using the ArcGIS for JavaScript Application Programming Interface and ArcGIS Online platform for exploration and projection of these data.

**Results:**

Literature searches yielded a total of 4,084 susceptibility data points for 1,505 populations, and 2,097 resistance mechanisms data points for 1,000 populations of *Anopheles spp.* tested via recommended WHO methods from 54 countries between 1954 and 2012. For the Afrotropical region, data were most abundant for populations of *An. gambiae*, and pyrethroids and DDT were more often used in susceptibility assays (51.1 and 26.8% of all reports, respectively) than carbamates and organophosphates. Between 2001 and 2012, there was a clear increase in prevalence and distribution of confirmed resistance of *An. gambiae s.l.* to pyrethroids (from 41 to 87% of the mosquito populations tested) and DDT (from 64 to 91%) throughout the Afrotropical region. Metabolic resistance mechanisms were detected in western and eastern African populations and the two *kdr* mutations (L1014S and L1014F) were widespread. For *An. funestus s.l.,* relatively few populations were tested, although in 2010–2012 resistance was reported in 50% of 10 populations tested. Maps are provided to illustrate the use of IR Mapper and the distribution of insecticide resistance in malaria vectors in Africa.

**Conclusions:**

The increasing pyrethroid and DDT resistance in *Anopheles* in the Afrotropical region is alarming. Urgent attention should be afforded to testing *An. funestus* populations especially for metabolic resistance mechanisms. IR Mapper is a useful tool for investigating temporal and spatial trends in *Anopheles* resistance to support the pragmatic use of insecticidal interventions.

## Background

Malaria remains one of the major disease burdens globally with over 207 million cases and 627,000 deaths estimated in 2012, mainly in children under 5 years old and predominantly in Africa [1]. The Democratic Republic of Congo (DRC) and Nigeria account for 40% of the total estimated global deaths, and combined with India account for 40% of the total estimated cases. Malaria is considered to be one of the major contributors to poverty and the estimated annual cost to economies of the African continent ranges from under 0.5% to almost 9% of GDP [2]. At 1% of the total African GDP of US$ 1,184 trillion, this translates into US$ 12 billion per annum. In addition to the scale of mortality and the loss of productivity due to illness, malaria also has devastating effects on cognitive development in children surviving the disease, leaving many disabled for life [3,4].

The dominant mosquito species responsible for the transmission of malaria parasites in Africa are mainly *Anopheles gambiae s.s., An. coluzzii* (formerly *An. gambiae s.s.* M form)*, An. arabiensis* and *An. funestus s.s.* that are widespread over tropical and subtropical Africa, although *An. arabiensis* prefers drier habitats and *An. coluzzii* is restricted to west-central Africa [5,6]. The adult behaviour and larval biology of these species are different, which impacts the ease with which each can be controlled. Adult *An. gambiae s.s., An. coluzzii* and *An. funestus s.s.* prefer feeding on humans and resting inside human habitations, while *An. arabiensis* will feed on either humans or cattle and rest indoors or outdoors making this vector more difficult to control [5-7]. *Anopheles funestus* prefers to breed in swampy, well-vegetated, permanent water bodies, while *An. gambiae s.s., An. coluzzii* and *An. arabiensis* can be found in temporary rain pools, hoof prints around the edges of dams and pans, and also in rice paddies [5-7]. Larviciding and/or larval habitat modification is therefore not always simple or feasible.

The most widespread and effective approach to controlling malaria vector mosquitoes is through the use of insecticide treated bed nets (LLINs) and indoor house spraying with residual insecticides (IRS). By far the most common insecticides used for house spraying are pyrethroids and this class of insecticide is the only one currently recommended by the World Health Organization (WHO) for treatment of bed nets [1]. It is therefore not surprising that malaria vectors have developed resistance to pyrethroids throughout the African continent [8-10]. Three other classes of WHO-recommended adulticides (organophosphates, carbamates and organochlorines) have also been used in IRS to differing extents throughout Africa, and resistance has been detected in *Anopheles* spp. to all three [11]. Insecticide resistance data are needed to inform vector control policy, particularly in the context of the Global Plan for Insecticide Resistance Management (GPIRM) that seeks to preserve or prolong the effectiveness of vector control interventions [11].

For optimal utility in informing policy, vector data need to be accurate, up-to-date, easily accessible and interpretable at operationally relevant scales. A number of papers have produced maps of malaria vector species on a country level [12-15] and across the African continent [6,9,10,16-18], mostly dealing with the distribution of the vectors but very few with insecticide resistance [8-10]. Methods used range in complexity, from simple presence/absence plotting on a map [8,9,12,16,17] to more sophisticated predictive models [17,19-23]. The difficulties facing interpretation of historical species occurrence records such as poor data coverage and taxonomic ambiguity of species also affect the mapping of insecticide resistance. Other issues facing the consolidation of insecticide resistance data include a bias towards reporting of susceptibility results only when resistance is subsequently detected [24,25], insufficient information to determine if standard protocols were adhered to, incorrect or missing geo-coordinates for collection sites, and a proliferation of published data for sentinel sites favoured by research groups while large volumes of data for other regions remain unpublished [9]. Disparities in criteria for reporting resistance also complicate matters - this is of particular relevance given a recent increase in the threshold for reporting resistance from 80% to 90% mortality in WHO susceptibility tests [26].

Static maps in publications quickly become outdated due to rapid changes in insecticide resistance status being reported in the current relatively high volume of publications on the topic. Until recently there was no consolidation of all historical and up-to-date information on insecticide resistance in malaria vectors. While there have been a number of initiatives to create online repositories specifically for such data, including within the Mapping Malaria Risk in Africa (MARA) [9] and VectorBase [27] databases, these platforms did not include quality control for uploaded data or failed to present summaries of recent data in a format appropriate for informing vector control decisions.

In this paper we introduce the free online geospatial application IR Mapper ( http://www.irmapper.com), which facilitates the exploration and projection of worldwide insecticide resistance data. We also provide an update on the status of insecticide resistance in the main malaria vectors in Africa including an historical review based on published reports from insecticide susceptibility tests and mechanisms investigations. Data are reviewed with respect to updated WHO guidelines refining the definition of “resistance” [26].

## Methods

### Insecticide resistance database

A systematic search of the published, peer-reviewed literature using online scientific bibliographic databases was performed using key words in English and French. Databases included PubMed [28], Web of Science [29], archives of MalariaWorld [30], Google Scholar [31], and the Armed Forces Pest Management Board [32]. Similarly, archives of 10 top journals in the field of *Anopheles* and insecticide research (Malaria Journal, Parasites and Vectors, PLOS ONE, Medical and Veterinary Entomology, Journal of Medical Entomology, Tropical Medicine and International Health, American Journal of Tropical Medicine and Hygiene, Transactions of the Royal Society of Tropical Medicine and Hygiene, Journal of Vector Ecology, Journal of Vector Borne Diseases) were searched. Key words used in singular or combination and in English and French were acetylcholinesterase, *Anopheles,* carbamate, DDT, esterase, GST, insecticide, *kdr*, pyrethroid, metabolic, mono-oxygenase, organochlorine, organophosphate, oxidase, resistance, resistant, target site, and names for individual countries. The search exercise was completed for all 54 countries of the African region. This process was similarly conducted for other countries outside Africa (17 to date).

Reference sections of all relevant located articles were also reviewed to identify additional literature. The dataset was augmented with three extra unpublished sources of information. The first was the African Network for Vector Resistance (ANVR) data extracted from IRBase [33]. The second was a summary report by the President’s Malaria Initiative (PMI) including WHO susceptibility test and *kdr* data from 18 countries [34]. The third source was unpublished reports of WHO susceptibility tests conducted using standard procedures on *Anopheles* populations from Cote d’Ivoire (2008, 2010), Democratic Republic of Congo (2007, 2011), Mali (2000, 2003, 2005, 2006, 2007, 2011) and Tanzania (2004, 2012) (Richard Hunt, unpublished data).

Data were extracted into Microsoft Excel 2010 (Redmond, USA) datasheets. For each insecticide susceptibility or mechanisms test conducted, the following were recorded: country name, locality name, GPS coordinates (latitude, longitude) of locality, mosquito collection period, vector species or species complex/group tested. Where data on mosquito collection period were not reported in the publication, efforts were made to contact the authors to obtain this information. In the event that no response was received from the authors, the year of publication was taken as the year of mosquito collection. For WHO susceptibility tests on adult mosquitoes, the following were recorded: insecticide type, insecticide class, Insecticide Resistance Action Committee’s (IRAC) mode of action classification and code, insecticide dosage, number of mosquitoes exposed per assay, measured percentage mortality corrected for controls, and susceptibility status. Status was assigned based on recently-revised WHO criteria [26], where: mortality <90% = confirmed resistance; 90-97% = possible resistance (with presence of resistant genes to be confirmed); 98-100% = susceptible. Where mortality rates were reported in range format, the average of the highest and lowest values was used to assign resistance status.

For molecular or biochemical mechanisms data, the following were recorded: test method used, mechanism tested for, number of mosquitoes used per assay, outcome of the assays (detected/ not detected), frequency of mutations. No assumptions were made in the data abstraction, with all reported data accurately reflecting the level of detail given in the data source. Geo-referencing was conducted in decimal degrees format using the set of coordinates provided in publications. Where coordinates were not listed, these were determined by locality names via Geonames [35] and failing that, Google Earth [36]. Where the administrative unit name was given only, this was included in the database with coordinates assigned based on the centre point of the unit; administrative units ranged from districts or counties to sub-districts or sub-counties depending on the particular publication. Data from localities for which coordinates could not be located were excluded from the database.

Data extractions primarily focused on publications with the assumption that published data were quality assured. However, some of the extracted data did not adhere to standard WHO protocol. For instance, in WHO susceptibility tests, assays were conducted using insecticide papers with non-standard concentrations of insecticide, or fewer than the recommended minimum of 100 mosquitoes were tested to derive a mortality rate. For this reason, such major parameters influencing study outcomes were also included in the database for presentation on the IR Mapper interface and were considered during the review process, as outlined below.

Following extraction, data were subjected to a two-level checking procedure, with the first level check conducted by a different abstractor to ensure an independent assessment of the assembled data. Data were cross-checked with any repeat records removed, especially for instances where unpublished PMI or ANVR data had subsequently been published. All aspects of the data were reviewed to ensure the information had been correctly collated and the sites geo-referenced accurately. The second level check incorporated suggested changes with an emphasis on geo-referencing. Third-level checks were implemented using the interface outlined below to identify: (i) inconsistent or non-standard spellings within fields that would affect filter query summaries; (ii) blanks in mandatory data fields; (iii) any geo-locations that fell outside the specified country boundaries or in the sea or other major water body; and (iv) any localities with similar names but different coordinates in order to harmonize naming/location data.

Literature searches and data extraction for the purpose of this review were concluded on 15 May 2013. However, the intention is for the process described above to continue on a monthly basis to ensure regular update of the database accessible via the IR Mapper online interface.

**Figure 1:**
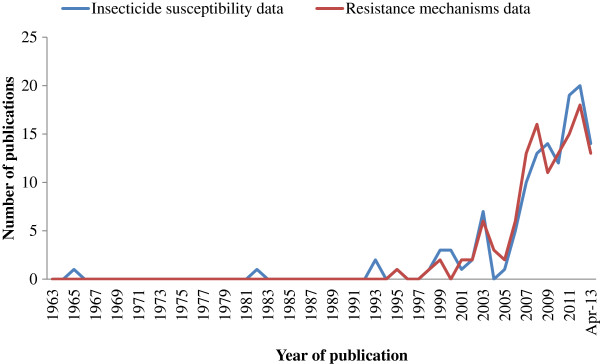
Number of publications from which data were extracted on insecticide susceptibility and resistance mechanisms, up to 30th April 2013.

The database contained information extracted from 224 individual publications from 60 peer-reviewed journals. There were relatively few publications up until the 1990s, with a rapid increase in both insecticide susceptibility and resistance mechanisms reported after 2005 (Figure 1). For the first four months of 2013, there were 14 publications that included data on susceptibility and 16 on mechanisms (18 in total). It is likely, therefore, that the number of publications for 2013 may exceed that of previous years (maximum of 26 in 2012). The database includes a total of 4,084 insecticide susceptibility tests for 1,505 *Anopheles* populations collected from 1954 to 2012, and 2,097 resistance mechanisms tests for 1,000 populations collected from 1987 to 2012.

### Online mapping interface

**Figure 2:**
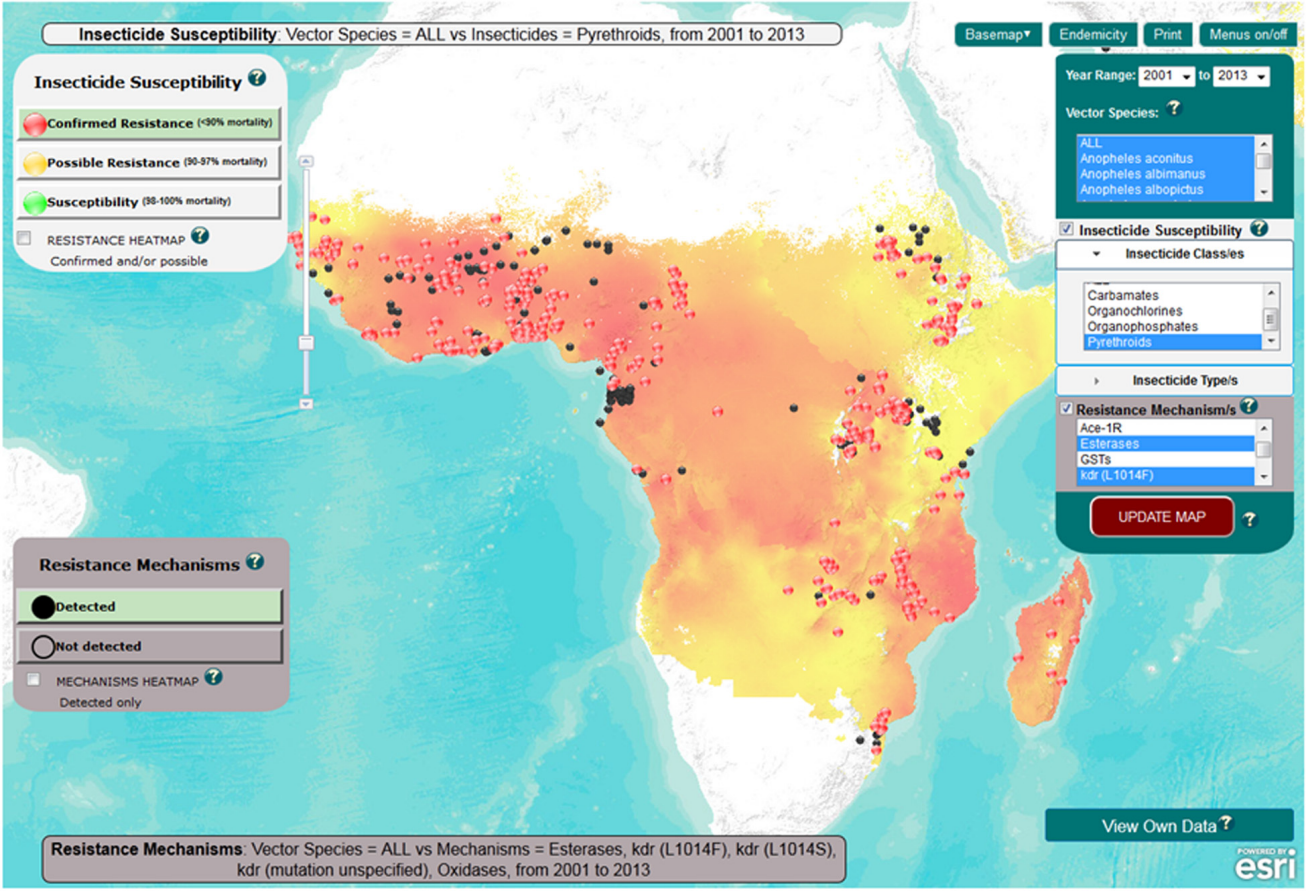
**IR Mapper online user interface showing *Anopheles spp. *** **pyrethroid resistance (red dots) and detected elevated mono-oxygenase and esterase activity plus *kdr *** **mutations (black dots) from 2001 – 2013 (as at 06/12/2013) along with *Plasmodium falciparum *** **endemicity estimates for 2010.**

An online interactive mapping platform, IR Mapper ( http://www.irmapper.com), was developed to allow users to project geo-referenced data residing in the database (Figure 2). The web application was built on the ArcGIS Application Programming Interface for JavaScript. Outcome data from WHO susceptibility tests (confirmed resistance, possible resistance, susceptible) and mechanisms assays (detected, not detected) are displayed. Details of assays (including insecticide dosages and numbers of mosquitoes tested) and links to data sources are provided in pop-up boxes associated with individual point data. The mapping application facilitates filtering of data via user-specified criteria for *Anopheles* species, insecticide class/es or type/s, resistance mechanism/s and dates of mosquito collections. A time filter enables users to view data by single or multiple year increments based on the start date of mosquito collections. *Plasmodium falciparum* and *Plasmodium vivax* endemicity maps [37,38] provided by the Malaria Atlas Project [39] are incorporated as optional layers. User-generated map images can be printed or saved.

Extended functionality allows users to download a standardised Excel template, add their own geo-referenced data and temporarily visualise these data on the mapping interface. Filters can then be applied to users’ own and existing data, and if desired the latter can be hidden via the left hand data layer menu. Therefore, this provides a facility for users to visualise (and print) all or part of their own unpublished data set either independently or alongside the published data set. Users’ data are not added to the online database and are cleared once the browser is closed. This ensures that all data available on the IR Mapper interface have undergone the quality control and assurance processes outlined above.

### Review of insecticide resistance status for the Afrotropical Region

**Figure 3:**
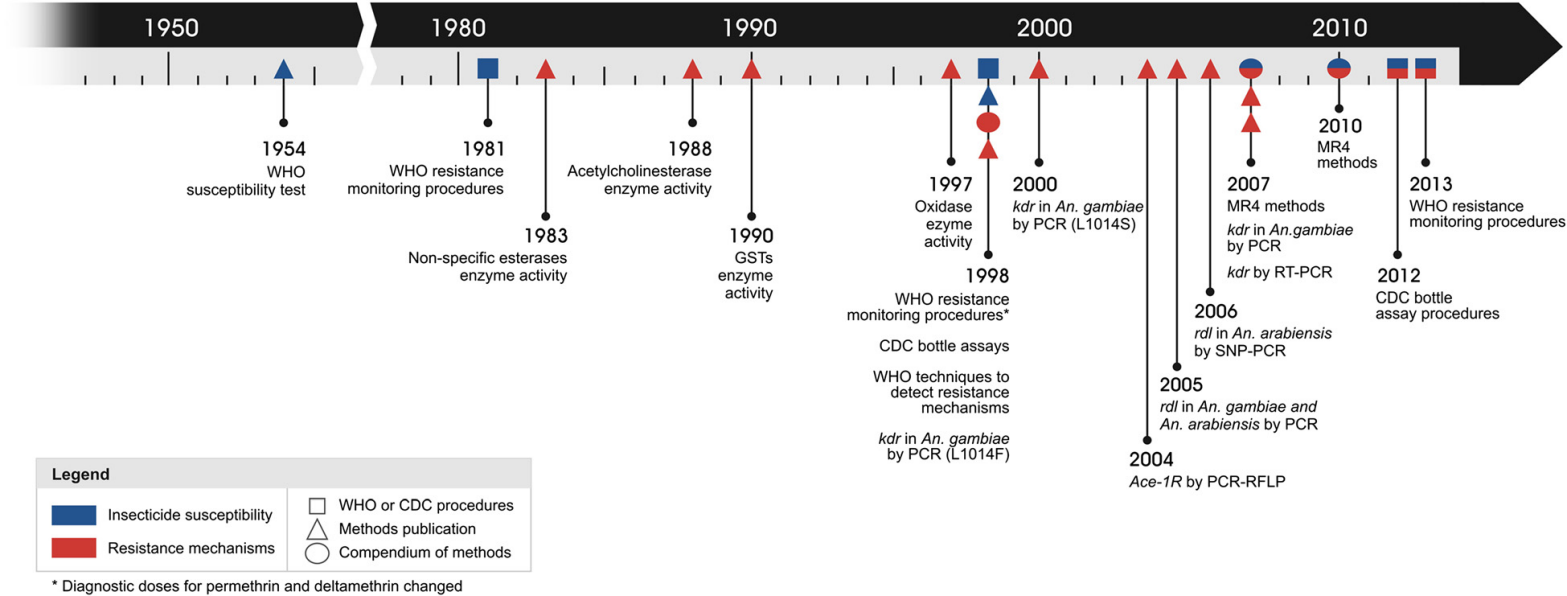
**Publication dates of key protocols and procedures used in insecticide resistance evaluations for *Anopheles*.** *Ace-1R*: insensitive acetylcholinesterase G119S mutation; CDC: Centres for Disease Control and Prevention; GSTs: elevated glutathione *s*-transferase; *kdr*: L1014S and L1014F target site mutations; MR4: Malaria Research and Reference Reagent Resource Centre; PCR: polymerase chain reaction; PCR-RFLP: PCR restriction fragment length polymorphism; *rdl*: dieldrin resistance-conferring chloride ion channel mutation; RT-PCR: reverse transcriptase PCR; SNP-PCR: single nucleotide polymorphism PCR; WHO: World Health Organization.

A sub-set of the full database described above and available on IR Mapper was extracted for the purpose of this review. Date of acceptance for publication was used as the cut-off, with data from articles accepted after 30th April 2013 excluded. PMI [34] and ANVR [33] unpublished datasets were excluded on the basis that data ownership resides with the institute/s responsible for collecting and reporting the data for their region. Results from WHO susceptibility tests with insecticidal papers that were not of standard discriminating dosages according to WHO [26] were excluded from the review, with the exception of permethrin and deltamethrin for which different standard dosages (0.25% and 0.025%, respectively) applied from 1981 [40] until 1998 [41] (Figure 3). Since discriminating dosages have yet to be defined for alpha-cypermethrin and chlorpyrifos-methyl, susceptibility data for these insecticides were excluded from the data set.

Scope was limited to tests on adults of the main African malaria vector species of the *An. gambiae* complex and *An. funestus* group, with reviews conducted based on four groupings: a) all species of the *An. gambiae* complex; b) *An. gambiae s.s.*; c) *An. arabiensis*; and d) all species of the *An. funestus* group. For the purpose of the review, *An. gambiae s.s.* (previously the S molecular form) and *An. coluzzii* (formerly *An. gambiae s.s.* M form) were grouped together since the nomenclature change is recent [5] and thus is yet to be consistently adhered to in publications. A population was defined as any species or species complex/group collected from one reported location and at one reported time period. The duration of collections varied by study, and was not always specified; these may have varied from days to months. For review purposes, aggregation of data by year was based on the start year of the collection period. Predicted distribution maps by vector species were sourced in GIFF format [42]; individual species maps were used for *An. gambiae s.s.* and *An. arabiensis* or maps of individual species were merged to create maps by *An. gambiae* complex or *An. funestus* group. All maps for the purposes of the review were created in ArcGIS for Desktop 10.1 (Esri, Redlands, CA).

## Results

The example screenshot of the IR Mapper online user interface shown in Figure 2 depicts recorded pyrethroid resistance (red dots) and detected elevated mono-oxygenase activity, elevated esterase activity, and/or *kdr* mutations (black dots) for *Anopheles* spp. populations collected from 2001 to 2013 (as at 06/12/2013). These point data are overlaid with a map layer showing estimated levels of *Plasmodium falciparum* malaria endemicity within the limits of stable transmission for 2010 [37].

Additional maps show the capacity for data residing in IR Mapper to be used to illustrate more detailed aspects such as temporal and spatial distribution in insecticide resistance and the known mechanisms underpinning resistance for each of the major malaria vectors or species groups.

### Susceptibility bioassays

Insecticide susceptibility data were recorded for 28 countries and resistance mechanisms data for 31 countries out of the 54 in the African region. Data on susceptibility were most numerous for Benin, Cameroon and Mozambique (92, 81 and 79 populations, respectively) and for mechanisms were the most numerous for Burkina Faso, Benin and Kenya (166, 128 and 102 populations, respectively). Both data types were most often reported for *Anopheles gambiae s.l.* (716 populations) with significantly fewer reports for members of the *An. funestus* group (123 populations); bioassay data were rarely reported for sibling species indicating how seldom molecular species differentiation was conducted for bioassay specimens (Additional files 1 and 2). The number of populations tested seemed to decrease in 2010–2012, but this is likely because results obtained for collections from this period are yet to be published. Across species and time, pyrethroids were generally the most tested insecticide class (51.1% of all test reports), followed by organochlorines (26.8%), carbamates (12.9%), and organophosphates (9.2%). Testing for *kdr* mutations was most common for *An. gambiae s.l.* with testing for metabolic mechanisms seldom conducted. For the period 2001–2012, the majority of insecticide susceptibility (78%) and resistance mechanism (73%) tests were reported for *Anopheles* populations from the 20 top malaria burden countries in Africa although relatively few data were available for some key countries such as DRC (Additional file 3).

During 2001–2012, resistance was detected to at least one insecticide in 78.5% (472/601) of *An. gambiae s.l.* populations from 27 of the 28 countries tested (with susceptibility only detected in Guinea Bissau) and in 43.6% (34/78) of *An. funestus* from 11 out of 13 countries tested (with susceptibility only detected in Burundi and Tanzania). Resistance to two classes of insecticides was detected in 219 populations of *An. gambiae s.l.* and 11 of *An. funestus* while resistance to three classes was found in 40 and 2 populations of these species, respectively. Resistance to insecticides from all four classes was detected in individual *An. gambiae s.l.* populations from five localities in Burkina Faso, three in Cote d’Ivoire and one each in Mali and Sudan. The highest numbers of reports of confirmed resistance since 2001 were for *Anopheles* populations from Benin (151), Cameroon (104) and Nigeria (94).

**Figure 4:**
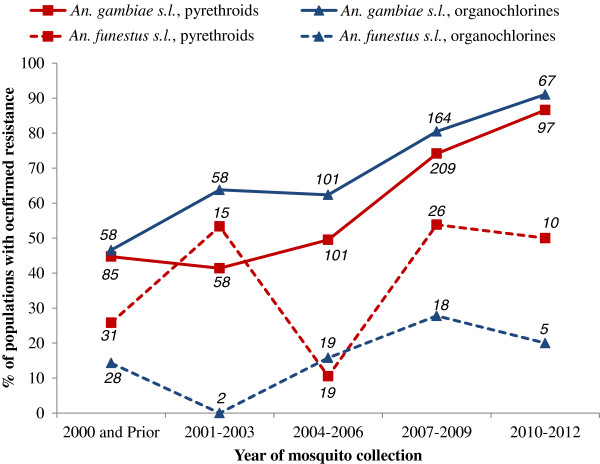
**Proportion of populations of *An. gambiae s.l. *and *An. funestus s.l. *with confirmed resistance using standard WHO susceptibility tests for pyrethroids and organochlorines.** Note that populations are accounted for more than once when tested with both insecticide classes. Italics indicate the number of populations tested.

The prevalence of *An. gambiae s.l.* resistance to pyrethroids and DDT increased between the periods 2001–2003 and 2010–2012: for pyrethroids from 41% to 87% and for DDT from 64 to 91% (Figure 4, Additional file 4). In *An. funestus*, pyrethroid resistance prevalence increased from 26% of 31 populations pre-2001 to 50% in 2010–2012 for 10 populations; a significant drop in 2004–2006 was observed though it is important to note that populations tested in the previous time period (2001–2003) were all from a single country (Mozambique). The prevalence of confirmed resistance of *An. funestus* to DDT was between 0% and 28%, though the lowest figure was for only two populations (Figure 4, Additional file 4). Similar examinations indicated no dramatic increases in the prevalence of carbamate or organophosphate resistance in either species group. There have been no reports of resistance of *An. funestus* to organophosphates since 1978.

### Resistance mechanisms

Investigations of resistance mechanisms in *An. gambiae* have largely focussed on target site mutations (*kdr* and *Ace-1*^*R*^); almost all populations tested for these mechanisms were differentiated to species, with the majority of populations identified as *An. gambiae s.s.* (M and S form) and the remainder as *An. arabiensis* (Additional file 5). Relatively few *An. gambiae s.l.* populations were assessed for metabolic mechanisms via biochemical assays; when conducted, the majority of metabolic testing was for mono-oxygenases and populations were often not identified to species. For *An. funestus*, testing for metabolic mechanisms and modified acetylcholinesterase enzyme activity was more prevalent prior to 2001, with only two *An. funestus* populations tested for *kdr* mutations since 2010.

**Figure 5:**
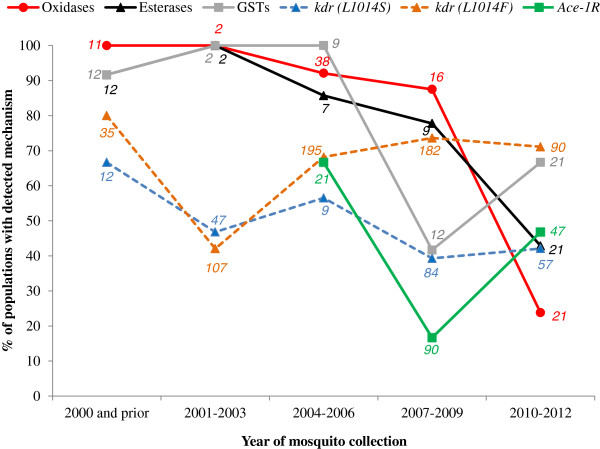
**Proportion of populations of *An. gambiae s.l. *with resistance mechanisms detected via standard biochemical or molecular methods.** Note that populations are accounted for more than once when tested for multiple resistance mechanisms. Italics indicate the number of populations tested.

The prevalence of L1014S in *An. gambiae s.l.* populations appeared to decrease after 2000 and then remain at moderate levels (39-56%) (Figure 5, Additional file 5). Conversely, the prevalence of L1014F populations appeared to increase after 2001–2003 and thereafter remain at higher levels (68 – 74%). While testing for metabolic mechanisms in *An. gambiae s.l.* was limited, the prevalence of elevated activity of mono-oxygenases, esterases and GSTs appeared to decline between the periods 2004–2006 and 2007–2009, with mono-oxygenases and esterases continuing to decline thereafter (to 24 and 43%, respectively) whereas detection of elevated GSTs became more common (67%). Testing for modified acetylcholinesterase was limited to few assessments via enzyme assays until methods for detecting the *Ace-1*^*R*^ mutation were developed, after which the gene was detected in 17 to 67% of *An. gambiae s.l.* populations depending on the period. No trends in the prevalence of resistance mechanisms were evident for *An. funestus* given the low number of populations tested.

### Distribution

**Figure 6:**
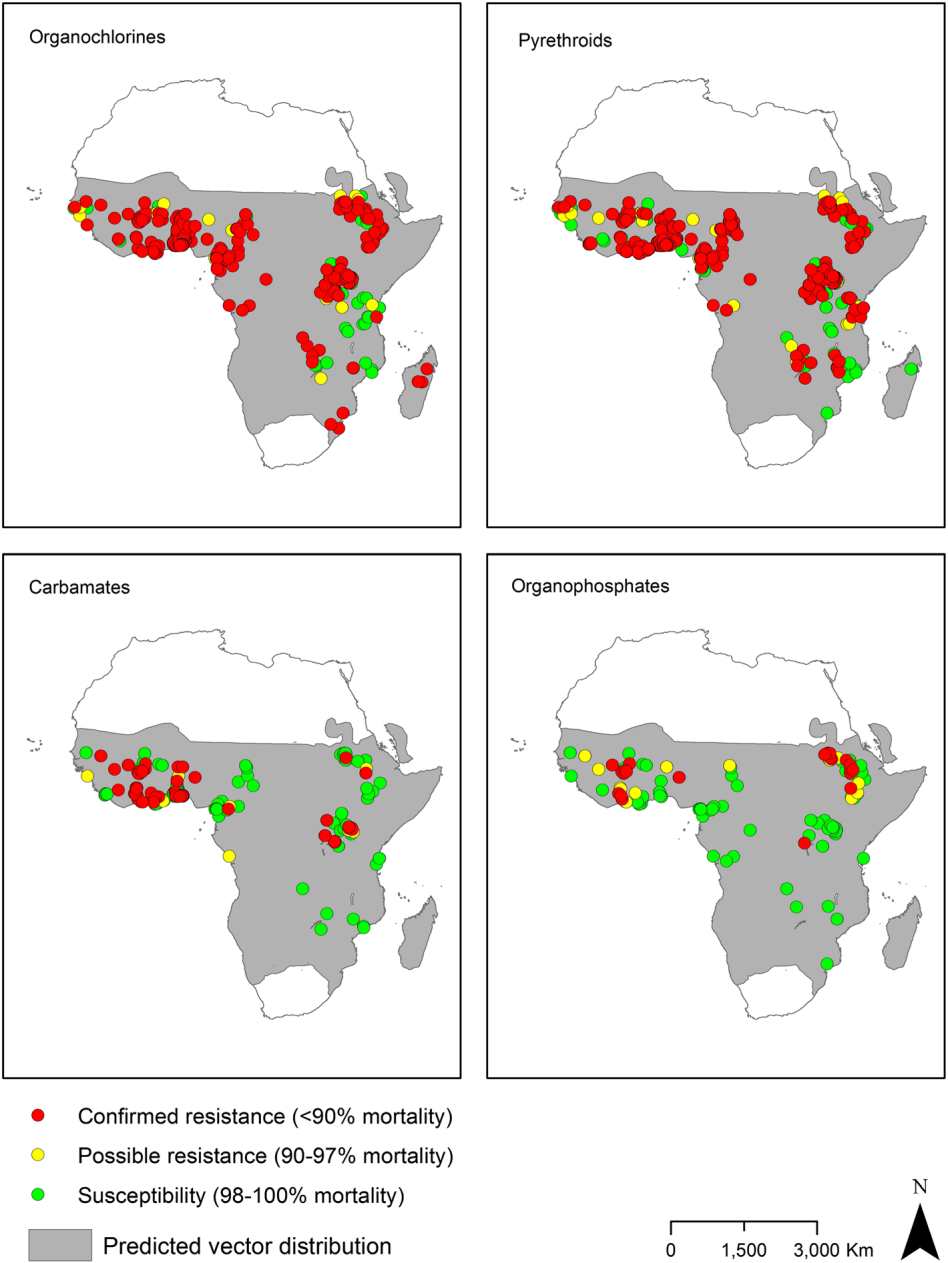
**Distribution of insecticide resistance in *An. gambiae s.l. *collected between 2001 and 2012.** Where there were multiple collections, species or insecticides tested, the lowest susceptibility category is displayed. Shading indicates the predicted distribution of the species complex [42].

**Figure 7:**
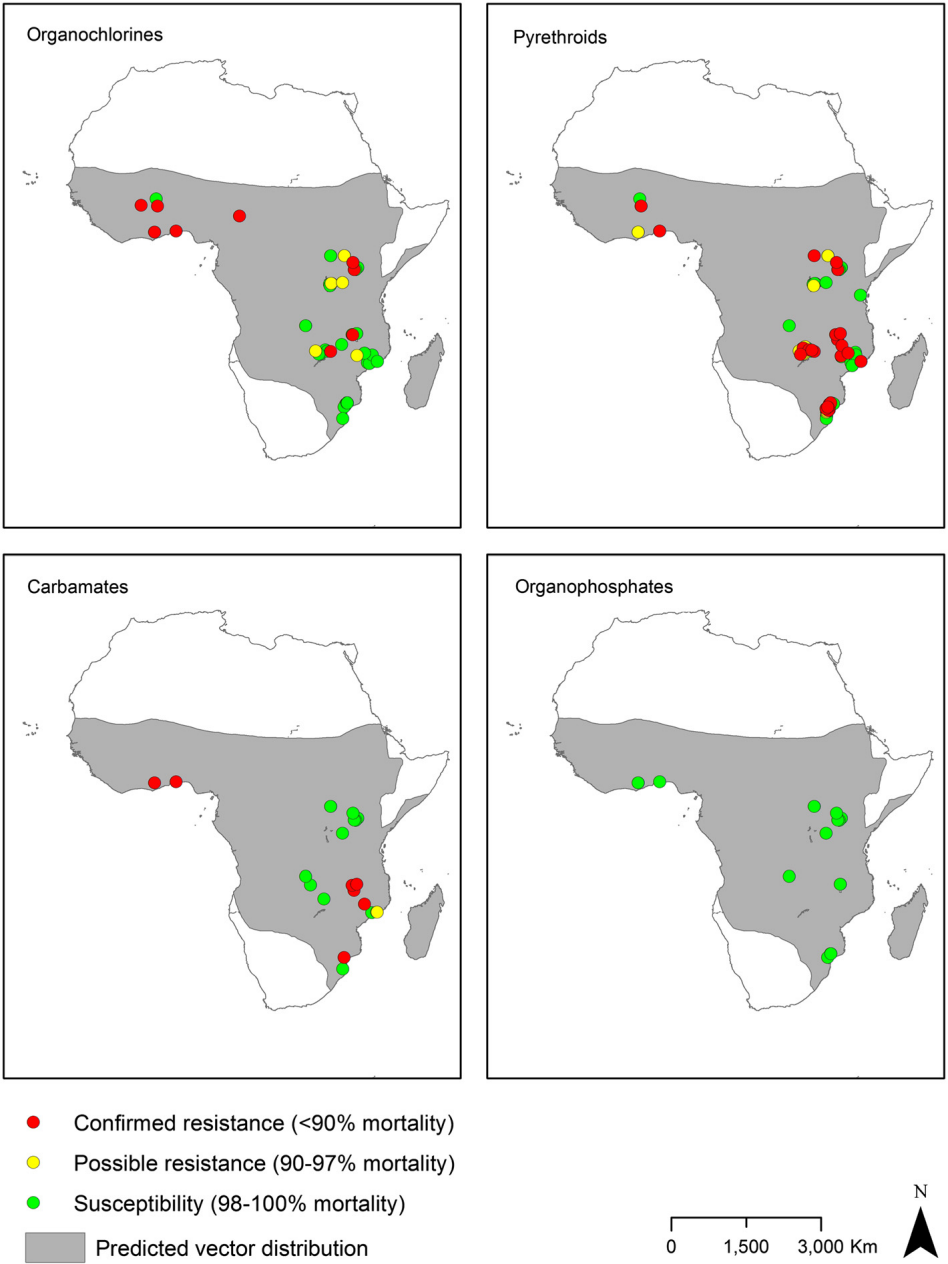
**Distribution of insecticide resistance in *An. funestus s.l. *collected between 2001 and 2012.** Where there were multiple collections, species or insecticides tested, the lowest susceptibility category is displayed. Shading indicates the predicted distribution of the species group [42].

**Figure 8:**
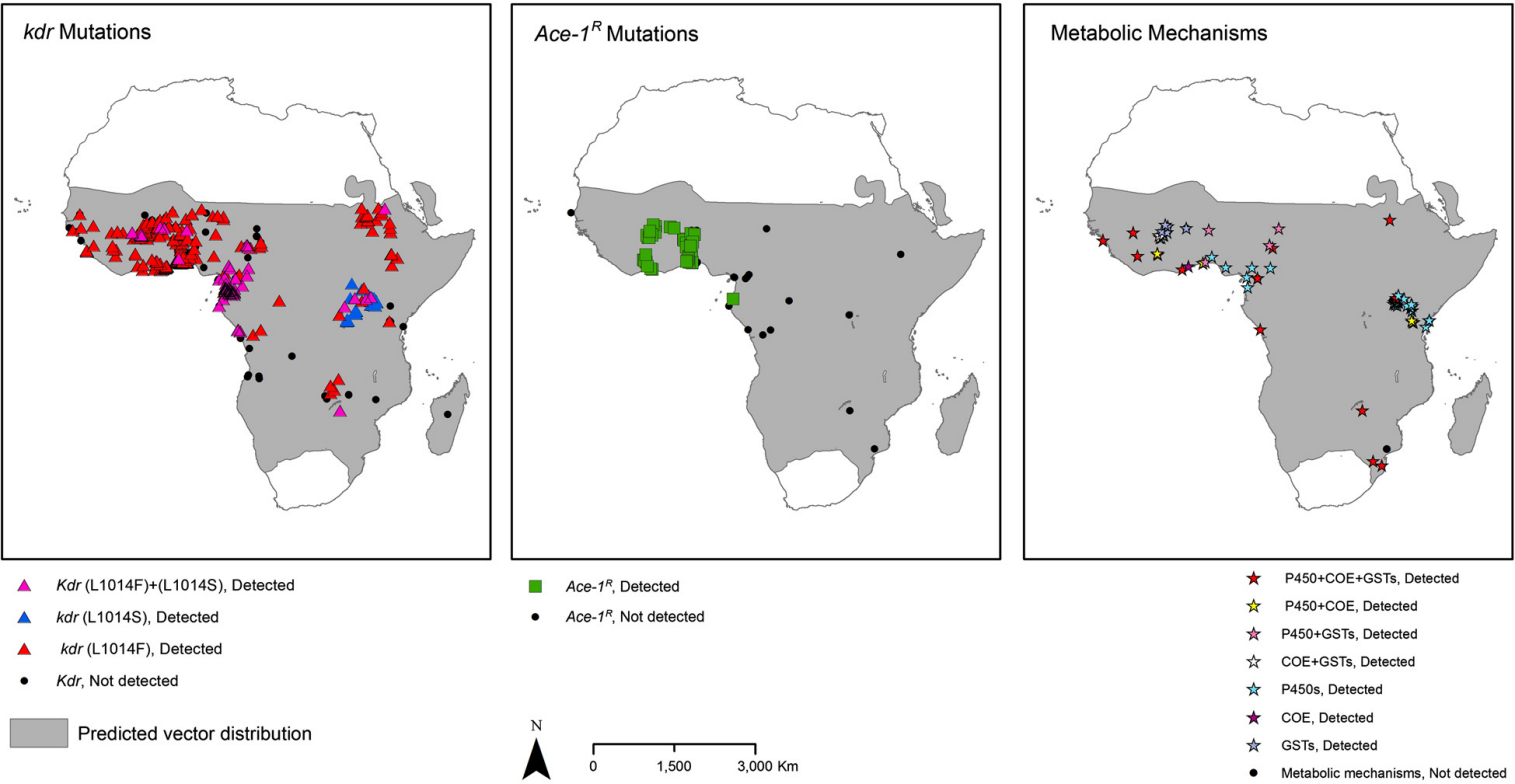
**Distribution of molecular / biochemical resistance mechanisms in *An. gambiae s.l. *collected between 2001 and 2012.** For sites for which multiple collections were tested, ‘detected’ is shown in preference to ‘not detected’. Shading indicates the predicted distribution of the species complex [42].

**Figure 9:**
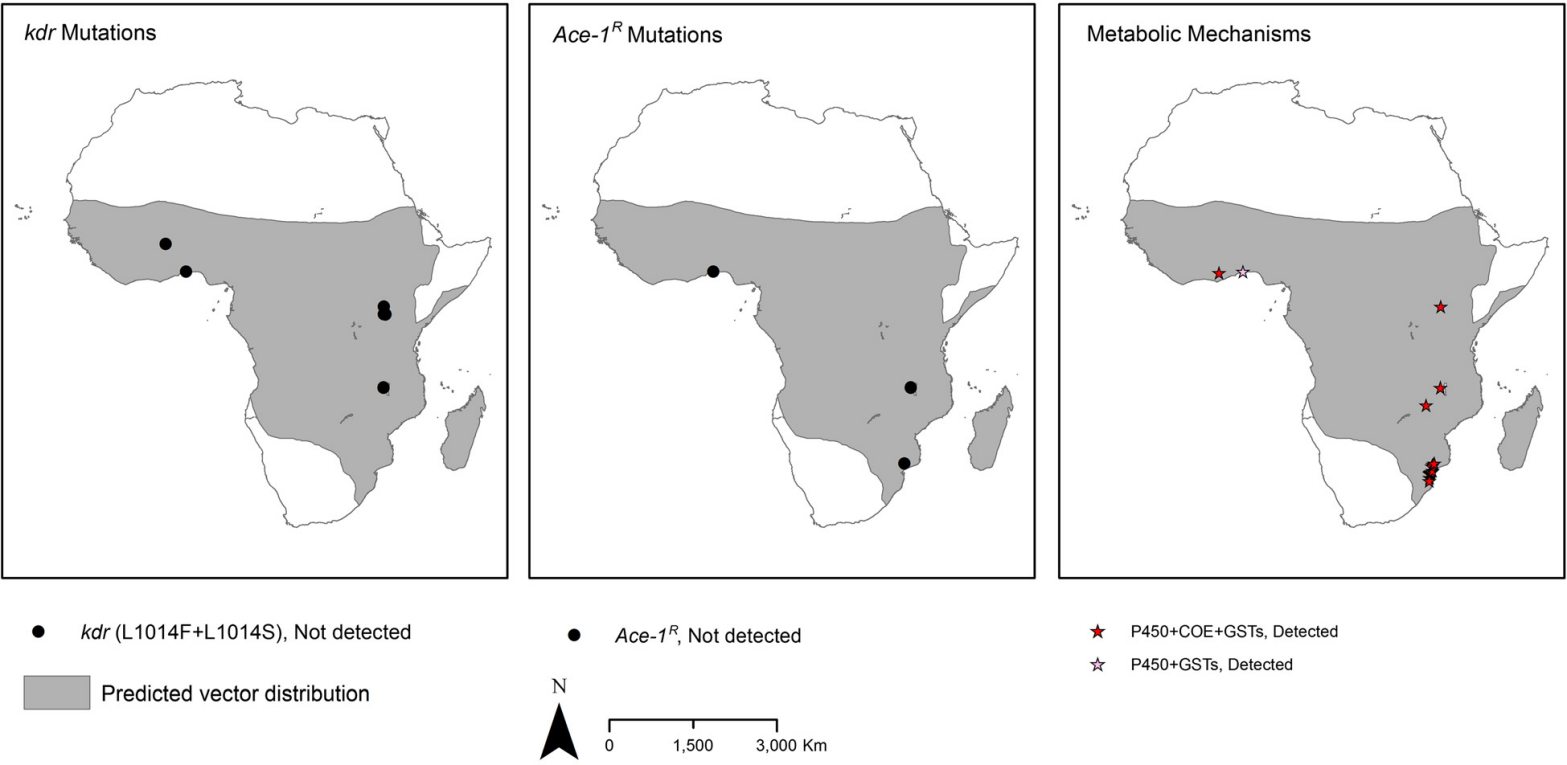
**Distribution of molecular / biochemical resistance mechanisms in *An. funestus s.l. *collected between 2001 and 2012.** For sites for which multiple collections were tested, ‘detected’ is shown in preference to ‘not detected’. Shading indicates the predicted distribution of the species group [42].

The spatial distributions between 2001 and 2012 of resistance to the four WHO-recommended adulticide classes and of the different resistance mechanisms are presented in Figures 6, 7, 8 and 9. These show that pyrethroid and DDT resistant *Anopheles* populations are widespread throughout the Afrotropical region, with foci of carbamate and organophosphate resistance reports in western Africa, Uganda, Ethiopia/northern Sudan and southern Africa. In terms of temporal trends in the geographic distribution of resistance, since 2001–2003 pyrethroid and DDT resistance have been reported in an increasing proportion of countries tested although this levelled off in 2010–2012 with resistance identified in 93% of the countries (Additional file 6). For *An. funestus*, populations have been tested in relatively few countries, confounding examination of the geographic spread of resistance. However, some spread in pyrethroid resistance is evident as prior to 2001 resistance was detected in one of four countries tested (Mozambique) whereas in 2010–2012 resistance was detected in the four countries tested (DRC, Kenya, Malawi, Mozambique).

Both *kdr* mutations (L1014S and L1014F) are widely distributed in *An. gambiae s.l.* throughout the Afrotropical region (Figures 8 and 9). Metabolic mechanisms are also widely distributed although few populations from central Africa have been tested. *Ace-1*^*R*^ has thus far been detected only in *Anopheles* populations from coastal areas of western Africa. Testing for resistance mechanisms in *An. funestus s.s.* has been sparse (Additional file 7).

Tailored maps featuring these data and the additional PMI [34] and ANVR [33] datasets can be created via the IR Mapper online application (Figure 2).

## Discussion

The number of scientific publications examining insecticide susceptibility and resistance mechanisms in *Anopheles* is growing rapidly. However, consolidated reviews of the status of resistance are few, and these can quickly become outdated due to the dynamic nature of insecticide resistance and the emergence of data for new or re-tested localities, insecticides or vector species. Main resistance status reviews were published for Africa by the ANVR in 2005 and 2012 [33,43], Coleman *et al*. in 2006 [9], Coetzee & Koekemoer in 2013 for *An. funestus*[44] and for pyrethroids by Ranson *et al*. in 2011 [10], with country profiles published by PMI [34] and an overview of recent (and often unpublished) data from malaria-endemic countries included in GPIRM [11]. These noted increasing resistance, but did not examine the full published historical data set for temporal and spatial trends.

The database displayed via the online geospatial application IR Mapper consists of data extracted from public documents, with monthly additions capturing information released in new publications. Review of a subset of the data from evaluations conducted on *Anopheles* from the Afrotropical region according to standard procedures [26] indicated that while there are data available for 28 African countries, testing is often concentrated in particular sentinel or study sites where appropriately skilled entomologists operate. These may not represent the most epidemiologically significant zones but rather may be favoured due to their proximity to research facilities. Due to this heterogeneous - and for many areas, sparse - distribution of resistance data, and also owing to the highly focalised and dynamic nature of insecticide resistance, producing region-wide estimates of resistance status may have limited utility for programmatic purposes. The exception may be if clear drivers of resistance, such as agricultural usage of insecticides, can be characterised and extrapolated for specific regions.

The high number of insecticide susceptibility reports for *An. gambiae s.l.* versus individual species of this complex is an indication of how seldom differentiation of sibling species is conducted post-bioassay. This is similarly the case for metabolic resistance mechanism assessments via biochemical assays. The paucity of data for individual members of the *An. gambiae* species complex and the *An. funestus* group limited comprehensive evaluations for these species and therefore this review focussed on wider species groupings. There is a critical need to identify specimens from susceptibility tests or mechanisms assays to species level, since insecticide susceptibility is clearly species-dependent. Further data are also required for *An. funestus*, which is a widespread and important vector especially in central and southern African countries but has been under-tested likely due to the relative difficulty of rearing adults from the progeny of wild blood-fed females or larval field collections. The lack of data for certain regions or species may also be due to a limited local capacity to conduct simple bioassays as well as propensity not to report data where widespread susceptibility is detected.

Resistance mechanisms testing for *An. gambiae* has largely focussed on target site mutations (*kdr* and insensitive acetylcholinesterase G119S [*Ace-1*^*R*^]) with relatively few resistant populations assessed for metabolic mechanisms. Evaluations of resistance mechanisms in *An. funestus* have been very sparse and mostly assessed metabolic mechanisms and acetylcholinesterase enzyme activity. Urgent attention should be afforded to testing for resistance mechanisms particularly in *An. funestus* populations given the emerging evidence on the relative importance of this species as a primary vector in an increasing number of countries in the Afrotropical region [44,45]. In particular, the focus should be on metabolic mechanisms, as it is generally accepted that metabolic-based resistance is likely to have more severe implications than target-site resistance [11]. Such information is crucial to guide evidence-based insecticide resistance management and deployment of the most appropriate tools.

The increasing prevalence and distribution of pyrethroid and DDT resistance in *An. gambiae s.l.* from the Afrotropical region is alarming. The GPIRM [11] was presented to deal with this, through the implementation of insecticide resistance management strategies aimed at preserving the efficacy of current tools. There is also a critical need to determine the impact that phenotypic resistance may be having on the efficacy of control tools across a range of eco-epidemiological settings, as well as factors contributing to such resistance and associations with the various resistance mechanisms.

Given our ongoing reliance on insecticidal interventions, it is likely that the necessity and demand for surveillance data will increase especially as concerns mount on the impact resistance may have on malaria vector control. In order to support informed deployment of insecticidal interventions, it is clear that resistance data must be collected, collated and fed back into the decision-making process in order to be optimally responsive to the local situation [26]. IR Mapper was primarily developed to collate essential data on insecticide resistance and make it readily available and easily digestible for malaria vector control programme managers and specialists. The tool allows rapid assessment of: (i) the geographic extent and frequency of resistance monitoring for specific vectors, insecticide classes, individual insecticides or mechanisms in a given region, and (ii) trends in resistance status and detected mechanisms over specified periods. Such information is used to identify major gaps in insecticide susceptibility and resistance mechanisms monitoring, as well as to ascertain the most up-to-date status based on published information. With the addition of users’ own data, unpublished data sets can also be included in such evaluations.

In terms of guiding interventions, while IR Mapper cannot directly inform product deployment, which depends on many other variables including price, availability, and suitability for intended end users, it does have a clear role in informing the selection of appropriate classes or insecticides for use in IRS as part of an evidence-driven insecticide resistance management strategy. The ability of IR Mapper to zoom in to targeted districts within a specific country provides the means for making large amounts of data far more easily comprehensible as well as highlighting regions where data are lacking. These visual maps are invaluable for monitoring trends in resistance development over time and planning insecticide-based control interventions accordingly. For instance, high resistance to pyrethroids (e.g. mortality in WHO susceptibility test of <50%) and a rapid increase in the frequency of *kdr* mutations in an area earmarked for IRS will suggest that an immediate switch to an acetylcholinesterase inhibitor such as a carbamate or organophosphate is justified as per GPIRM technical recommendations [11]. Alternatively, if susceptibility remains and *kdr* mutations are absent or frequency is stable, it may indicate that pyrethroids can continue to be used if applied pragmatically in consideration of the principals of pre-emptive resistance management. IR Mapper may also be used to advocate for increased monitoring in regions where specific issues of high resistance have been identified and also to determine if there are any changes over time in either insecticide susceptibility or resistance gene frequency following specific interventions.

Data contained on the IR Mapper platform should be interpreted with consideration of the limitations of the methods applied to derive the displayed data. WHO insecticide susceptibility and resistance mechanisms data cannot be used to draw conclusions about possible operational control failures of insecticidal vector control tools such as LLINs or IRS. For this, comprehensive field evaluations correlated with malaria case data would need to be conducted. Moreover, since IR Mapper does not allow for filtering of data on the basis of assay parameters such as insecticidal dosage on impregnated test papers, or the number of mosquitoes tested, users should be cautious in interpreting the data *en mass*. Relevant information is presented for each individual data point as is an online link to the original data source, and the onus is on the user to ascertain the relative importance of available data for guiding evidence-based surveillance and control strategies.

Although IR Mapper is currently limited to data from public scientific publications and a few other credible data sources (e.g. PMI, ANVR), incorporation of unpublished data sets is feasible if a robust verification and authorisation process in established. This would require a sustained input of labour considering the increase in volume of insecticide resistance data becoming available. Results from microarray studies or for newly-identified resistance mechanisms (such as new sodium channel mutations) can easily be incorporated on the existing configuration, since the user interface automatically reflects the parameters included in the database as long as the outputs of detected/not detected are defined. There is also the possibility to extend the platform for display of CDC bottle bioassay data [46], which may be important considering this is now being applied as the primary insecticide susceptibility surveillance method in some countries.

## Conclusions

IR Mapper is a simple tool for investigating temporal and spatial trends in *Anopheles* insecticide susceptibility and resistance mechanisms. National malaria control programs can use the platform to gain an overview of the spatial distribution and extent of available data to inform surveillance strategies. Outputs can be used to optimise malaria vector control via pragmatic use of insecticidal interventions and design of insecticide resistance management strategies. It is critical that such evidence-based approaches are prioritised given the apparent trend of increasing pyrethroid and DDT resistance in *Anopheles* in the Afrotropical region.

## Abbreviations

*Ace-1*^*R*^: Insensitive acetylcholinesterase G119S mutation; AChE: Acetylcholinesterase; ANVR: African network on vector resistance; CDC: Centre for disease control; DDT: Dichlorodiphenyltrichloroethane; DRC: Democratic Republic of Congo; GIFF: Graphics interchange format; GIS: Geographic information systems; GPIRM: Global plan for insecticide resistance management; GPS: Global positioning system; GST: Glutathione *s*-transferase; IRAC: Insecticide resistance action committee; IR: Insecticide resistance; IRS: Indoor residual spraying; *kdr*: L1014F or L1014S knockdown resistance mutation; KEMRI: Kenya Medical Research Institute; LLIN: Long-lasting insecticidal net; MAP: Malaria Atlas Project; MARA: Mapping malaria risk in Africa; NRF: National Research Foundation; PMI: President’s Malaria Initiative; WHO: World Health Organization.

## Competing interests

During the formulation of this tool and manuscript, TBK, EOJ and HPJ worked for Vestergaard Frandsen, a developer and manufacturer of insecticidal vector control tools. LN and PC worked for Esri Eastern Africa, a distributor of ArcGIS products. All other authors declare that they have no competing interests.

## Authors’ contributions

EOJ, MC, RNC, RHH and RN sourced data publications and reports. EOJ was responsible for primary data extraction, and EOO and TK conducted data verifications for the database. TBK, EOJ and HPJ conceived of the IR Mapper application and coordinated its development with LN and PC. TBK, EOJ and MC conceived of the review and devised the methodology. TBK and EOJ conducted the data analyses and mapping. All authors contributed to drafting the manuscript and all read and approved the final version.

## Supplementary Material

Additional file 1 **Number of populations for which resistance was confirmed and not confirmed using standard WHO insecticide susceptibility tests on adult mosquitoes.** A) *An. gambiae s.l.*; B) *An. gambiae s.s*.; C) *An. arabiensis*; and D) *An. funestus s.l.* by year of mosquito collection and insecticide class. Insecticide susceptibility testing was most common for *An. gambiae s.l.* populations. Pyrethroids and organochlorines were the most frequently tested insecticide classes. Click here for file

Additional file 2 **Number of populations for which resistance mechanisms were detected and not detected.** A) *An. gambiae s.l.*, B) *An. gambiae s.s*., C) *An. arabiensis* and D) *An. funestus s.l.* by year of mosquito collection and mechanism class. *An. gambiae s.l.* was commonly tested for mechanisms while *An. funestus s.l.* were not. *kdr* mutations were the most frequently tested resistance mechanisms in *An. gambiae s.l.* with metabolic mechanisms seldom tested. Click here for file

Additional file 3 **Number of *Anopheles *** **populations for which insecticide susceptibility and resistance mechanisms tests were conducted between 2001 and 2012 for top 20 malaria burden countries.** Few or no *Anopheles* populations were tested for insecticide susceptibility or resistance mechanisms in some of the countries with the highest malaria burden. Click here for file

Additional file 4 **Summary of data from WHO insecticide susceptibility tests conducted with *Anopheles spp. *** **populations collected in Africa between 1963 and 2012.** Prevalence of confirmed pyrethroid and organochlorine resistance in *An. gambiae s.l.* increased over time. Click here for file

Additional file 5 **Summary of data from resistance mechanisms tests conducted with *Anopheles spp. *** **populations collected in Africa between 1987 and 2012.** Prevalence of L1014S remained at moderate levels after 2001–2003 whereas prevalence of L1014F was at comparatively higher levels. Click here for file

Additional file 6 **Number of countries in Africa for which resistance was confirmed in at least one population of *Anopheles spp. *** **via WHO insecticide susceptibility tests.** Since 2001–2003, pyrethroid and DDT resistance have been reported in an increasing proportion of countries tested although this levelled off in 2010–2012. Click here for file

Additional file 7 **Number of countries in Africa for which resistance mechanisms were detected in at least one population of *Anopheles spp.*** Testing for resistance mechanisms in *An. funestus s.s.* has been sparse. Click here for file
