# Supplementary material for: An online tool for mapping insecticide resistance in major Anopheles vectors of human malaria parasites and review of resistance status for the Afrotropical region
Source: Parasit Vectors. 2014 Feb 21;7:76. doi: 10.1186/1756-3305-7-76 (PMC3942210; doi:10.1186/1756-3305-7-76)
Supplement: Additional file 1 — Number of populations for which resistance was confirmed and not confirmed using standard WHO insecticide susceptibility tests on adult mosquitoes. A) An. gambiae s.l.; B) An. gambiae s.s.; C) An. arabiensis; and D) An. funestus s.l. by year of mosquito collection and insecticide class. Insecticide susceptibility testing was most common for An. gambiae s.l. populations. Pyrethroids and organochlorines were the most frequently tested insecticide classes. [file 1756-3305-7-76-S1.pdf]

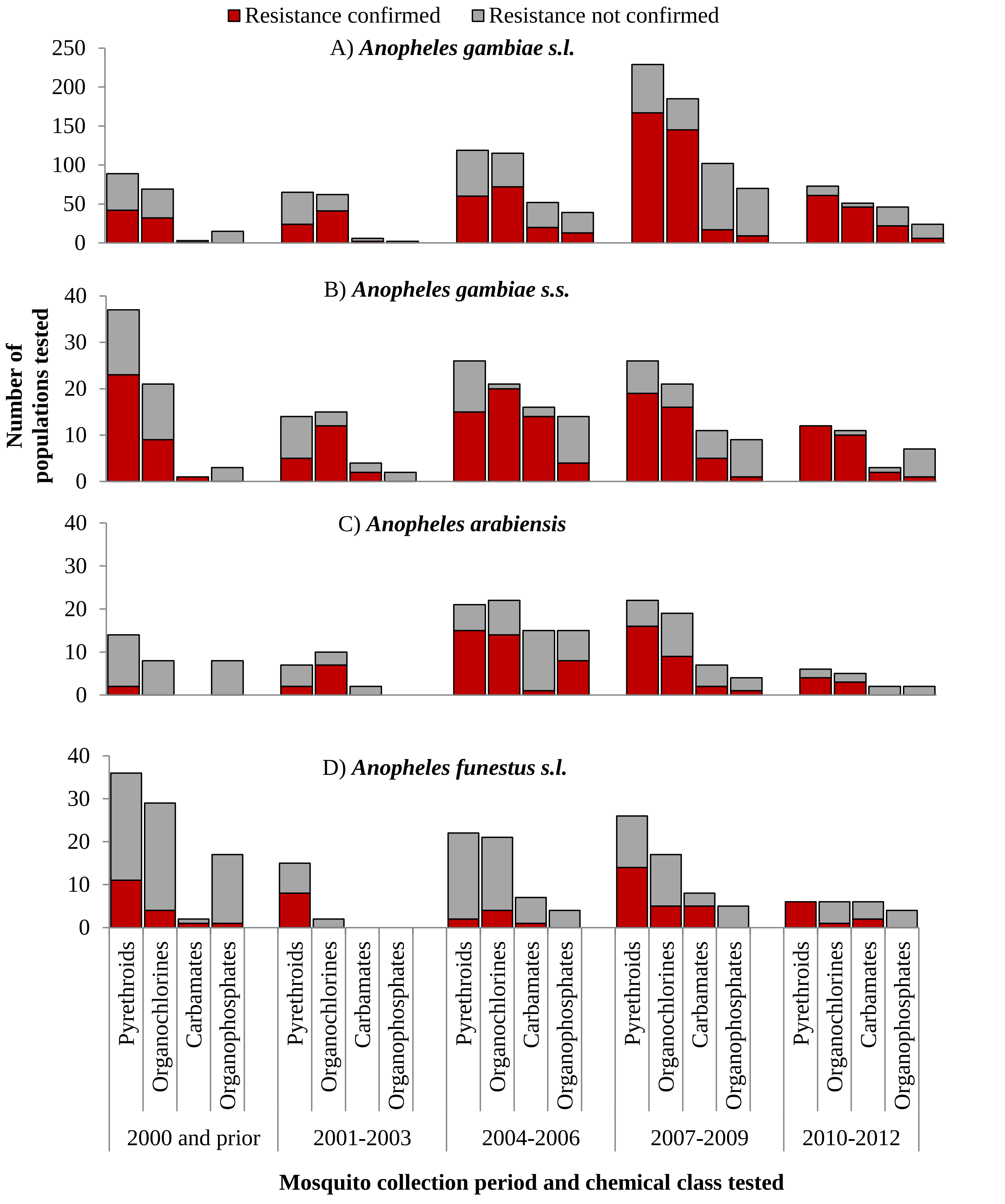

Number of populations for which resistance was confirmed (red) and not confirmed (grey) using standard WHO insecticide susceptibility tests [35] on adult mosquitoes. A) *An. gambiae s.l.*; B) *An. gambiae s.s.*; C) *An. arabiensis*; and D) *An. funestus s.l.* by year of mosquito collection and insecticide class.
