# Supplementary material for: An online tool for mapping insecticide resistance in major Anopheles vectors of human malaria parasites and review of resistance status for the Afrotropical region
Source: Parasit Vectors. 2014 Feb 21;7:76. doi: 10.1186/1756-3305-7-76 (PMC3942210; doi:10.1186/1756-3305-7-76)
Supplement: Additional file 2 — Number of populations for which resistance mechanisms were detected and not detected. A) An. gambiae s.l., B) An. gambiae s.s., C) An. arabiensis and D) An. funestus s.l. by year of mosquito collection and mechanism class. An. gambiae s.l. was commonly tested for mechanisms while An. funestus s.l. were not. kdr mutations were the most frequently tested resistance mechanisms in An. gambiae s.l. with metabolic mechanisms seldom tested. [file 1756-3305-7-76-S2.pdf]

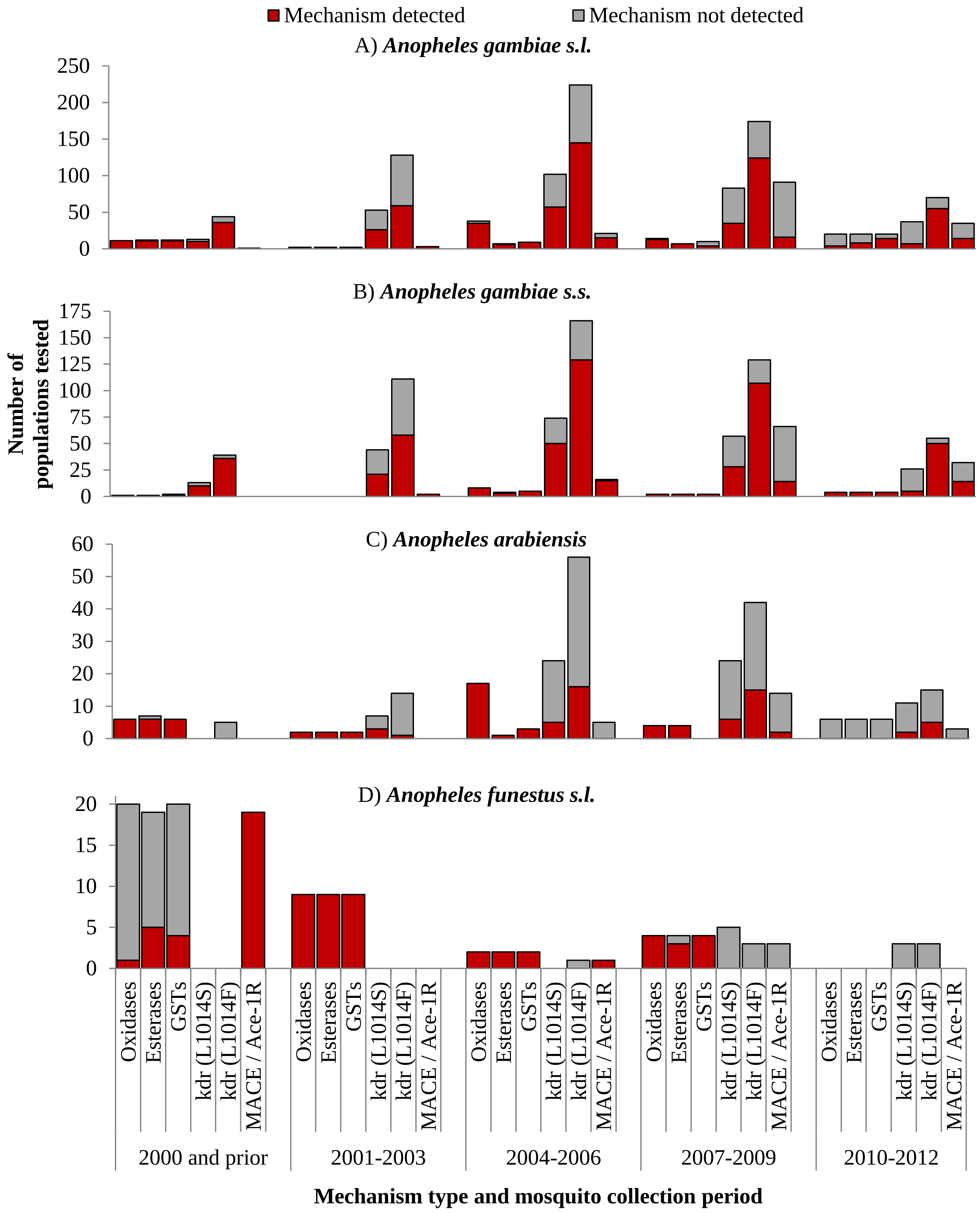

Number of populations for which resistance mechanisms were detected (red) and not detected (grey). A) *An. gambiae s.l.*, B) *An. gambiae s.s.*, C) *An. arabiensis* and D) *An. funestus s.l.* by year of mosquito collection and mechanism class.
