# Supplementary material for: An online tool for mapping insecticide resistance in major Anopheles vectors of human malaria parasites and review of resistance status for the Afrotropical region
Source: Parasit Vectors. 2014 Feb 21;7:76. doi: 10.1186/1756-3305-7-76 (PMC3942210; doi:10.1186/1756-3305-7-76)
Supplement: Additional file 3 — Number of Anopheles populations for which insecticide susceptibility and resistance mechanisms tests were conducted between 2001 and 2012 for top 20 malaria burden countries. Few or no Anopheles populations were tested for insecticide susceptibility or resistance mechanisms in some of the countries with the highest malaria burden. [file 1756-3305-7-76-S3.pdf]

Number of *Anopheles* populations for which insecticide susceptibility and resistance mechanisms tests were conducted between 2001 and 2012.

Country*	Number of populations tested since 2001	
	Insecticide susceptibility	Resistance mechanisms
Nigeria	72	78
Democratic Republic of Congo	9	5
United Republic of Tanzania	32	6
Uganda	38	20
Mozambique	33	10
Cote d'Ivoire	20	14
Sudan	33	18
Ghana	13	37
Burkina Faso	40	118
Ethiopia	24	4
Cameroon	81	72
Chad	14	6
Malawi	19	2
Niger	0	29
Angola	0	8
Guinea	4	5
Mali	9	9
Kenya	30	90
Zambia	24	7
Senegal	2	7
**Total**	**497**	**545**

*Top 20 countries according to highest reported malaria cases from WHO World Malaria Report[1]
