# Supplementary material for: An online tool for mapping insecticide resistance in major Anopheles vectors of human malaria parasites and review of resistance status for the Afrotropical region
Source: Parasit Vectors. 2014 Feb 21;7:76. doi: 10.1186/1756-3305-7-76 (PMC3942210; doi:10.1186/1756-3305-7-76)
Supplement: Additional file 4 — Summary of data from WHO insecticide susceptibility tests conducted with Anopheles spp. populations collected in Africa between 1963 and 2012. Prevalence of confirmed pyrethroid and organochlorine resistance in An. gambiae s.l. increased over time. [file 1756-3305-7-76-S4.pdf]

Number of *Anopheles spp.* populations collected in Africa between 1963 and 2012 with resistance confirmed or not confirmed via WHO insecticide susceptibility tests [26][§].

	**Time period^**	**2000 and prior**		**2001 - 2003**		**2004 - 2006**		**2007 - 2009**		**2010 - 2012**	
**Species or complex**	**Insecticide class**	*RC*	*NC*	*RC*	*NC*	*RC*	*NC*	*RC*	*NC*	*RC*	*NC*
An. gambiae s.l.*	Pyrethroids	38	47	24	34	50	51	155	54	84	13
	Organochlorines	27	31	37	21	63	38	132	32	61	6
	Carbamates	1	2	2	4	17	30	16	83	28	39
	Organophosphates	0	15	0	2	13	25	8	55	8	29
An. gambiae s.s.	Pyrethroids	20	12	5	9	14	11	28	7	13	0
	Organochlorines	9	12	10	3	18	1	16	5	11	1
	Carbamates	1	0	2	2	13	2	5	14	3	1
	Organophosphates	0	3	0	2	4	10	1	8	1	7
An. arabiensis	Pyrethroids	2	13	2	4	15	6	15	7	7	3
	Organochlorines	0	9	7	3	14	8	9	10	6	3
	Carbamates	0	0	0	2	1	14	2	6	1	8
	Organophosphates	0	8	0	0	8	7	1	5	2	6
An. funestus s.l.*	Pyrethroids	8	23	8	7	2	17	14	12	5	5
	Organochlorines	4	24	0	2	3	16	5	13	1	4
	Carbamates	1	2	0	0	1	6	5	3	2	4
	Organophosphates	1	16	0	0	0	4	0	6	0	3

[§] Data sources and extraction, compilation and verification processes are outlined in Methods section
[#] Includes all species within the complex plus non-differentiated species
^ Refers to year of commencement of field collections
RC = number of populations of the species/complex for which resistance was confirmed [26] to at least one insecticide of the specified class
NC = number of populations of the species/complex for which resistance was not confirmed [ref] to at least one insecticide of the specified class
