# Supplementary material for: An online tool for mapping insecticide resistance in major Anopheles vectors of human malaria parasites and review of resistance status for the Afrotropical region
Source: Parasit Vectors. 2014 Feb 21;7:76. doi: 10.1186/1756-3305-7-76 (PMC3942210; doi:10.1186/1756-3305-7-76)
Supplement: Additional file 5 — Summary of data from resistance mechanisms tests conducted with Anopheles spp. populations collected in Africa between 1987 and 2012. Prevalence of L1014S remained at moderate levels after 2001–2003 whereas prevalence of L1014F was at comparatively higher levels. [file 1756-3305-7-76-S5.pdf]

Number of *Anopheles spp.* populations collected in Africa between 1987 and 2012 with resistance mechanisms detected or not detected [26][§].

	**Time period^**	**2000 and prior**		**2001 - 2003**		**2004 - 2006**		**2007 - 2009**		**2010 - 2012**	
**Species or complex**	**Insecticide class**	*D*	*ND*	*D*	*ND*	*D*	*ND*	*D*	*ND*	*D*	*ND*
An. gambiae s.l.*	Oxidases	11	0	2	0	35	3	14	2	5	16
	Esterases	11	1	2	0	6	1	7	2	9	12
	GSTs	11	1	2	0	9	0	5	7	14	7
	kdr (L1014S)	8	4	22	25	52	40	33	51	24	33
	kdr (L1014F)	28	7	45	62	133	62	134	48	64	26
	Ace-1R	0	0	0	0	14	7	15	75	22	25
An. gambiae s.s.	Oxidases	1	0	0	0	8	0	2	0	5	0
	Esterases	1	0	0	0	3	1	2	0	5	0
	GSTs	1	1	0	0	5	0	2	0	4	1
	kdr (L1014S)	8	2	17	21	46	20	25	32	6	21
	kdr (L1014F)	28	2	44	46	115	23	116	22	55	5
	Ace-1R	0	0	0	0	14	2	13	47	21	16
An. arabiensis	Oxidases	6	0	2	0	17	0	5	1	0	6
	Esterases	6	1	2	0	1	0	4	2	0	6
	GSTs	6	0	2	0	3	0	1	1	0	6
	kdr (L1014S)	0	0	3	4	4	18	7	18	4	12
	kdr (L1014F)	0	5	1	13	18	37	16	25	9	10
	Ace-1R	0	0	0	0	0	5	2	15	0	6
An. funestus s.l.*	Oxidases	1	19	9	0	2	0	5	0	0	0
	Esterases	5	14	9	0	2	0	3	1	0	0
	GSTs	4	16	9	0	0	0	4	0	0	0
	kdr (L1014S)	0	0	0	0	0	0	0	5	0	3
	kdr (L1014F)	0	0	0	0	0	1	0	3	0	3
	Ace-1R	0	0	0	0	0	0	0	3	0	0

[§] Data sources and extraction, compilation and verification processes are outlined in Methods section

[#] Includes all species within the complex plus non-differentiated species

^ Refers to year of commencement of field collections

D = number of populations of the species/complex for which the specified mechanism was detected [26]. For target site mechanisms this refers to detection of the specified alleles; for metabolic mechanisms this refers to field populations exhibiting significantly higher enzyme activity and/or gene expression levels than that of the reference susceptible strain (using biochemical assays and/or detox chip microarrays).

ND = number of populations of the species/complex for which specified mechanisms was tested for but was not detected [26]
