# Supplementary material for: An online tool for mapping insecticide resistance in major Anopheles vectors of human malaria parasites and review of resistance status for the Afrotropical region
Source: Parasit Vectors. 2014 Feb 21;7:76. doi: 10.1186/1756-3305-7-76 (PMC3942210; doi:10.1186/1756-3305-7-76)
Supplement: Additional file 6 — Number of countries in Africa for which resistance was confirmed in at least one population of Anopheles spp. via WHO insecticide susceptibility tests. Since 2001–2003, pyrethroid and DDT resistance have been reported in an increasing proportion of countries tested although this levelled off in 2010–2012. [file 1756-3305-7-76-S6.pdf]

Number of countries in Africa for which resistance was confirmed in at least one population of *Anopheles spp.* via WHO insecticide susceptibility tests [26] [§].

	**Time period^**	**2000 and prior**		**2001 - 2003**		**2004 - 2006**		**2007 - 2009**		**2010 - 2012**	
**Species or complex**	**Insecticide class**	*RC*	*NC*	*RC*	*NC*	*RC*	*NC*	*RC*	*NC*	*RC*	*NC*
An. gambiae s.l.*	Pyrethroids	8	5	4	3	11	5	21	1	14	1
	Organochlorines	6	5	2	7	11	4	20	1	13	1
	Carbamates	1	1	1	2	5	5	9	8	9	5
	Organophosphates	0	3	0	1	4	6	4	13	3	9
An. gambiae s.s.	Pyrethroids	3	2	3	0	4	2	7	1	7	0
	Organochlorines	2	2	3	0	6	0	7	0	5	0
	Carbamates	1	0	1	1	4	1	4	3	2	1
	Organophosphates	0	2	0	1	2	3	1	5	1	3
An. arabiensis	Pyrethroids	1	1	2	1	3	2	6	2	4	1
	Organochlorines	0	2	3	1	4	0	4	3	3	1
	Carbamates	0	0	0	2	1	1	2	3	1	3
	Organophosphates	0	0	0	0	1	1	1	3	1	2
An. funestus s.l.*	Pyrethroids	1	4	1	0	2	6	5	2	4	0
	Organochlorines	2	5	0	1	2	7	5	3	1	3
	Carbamates	1	1	0	0	1	4	3	3	2	3
	Organophosphates	1	3	0	0	0	3	0	4	0	3

[§] Data sources and extraction, compilation and verification processes are outlined in Methods section

[#] Includes all species within the complex plus non-differentiated species

^ Refers to year of commencement of field collections

RC = number of countries for which at least one population of the species/complex had confirmed resistance [26] to at least one insecticide of the specified class

NC = number of countries for which no tested population had confirmed resistance [26] to at least one insecticide of the specified class
