# Supplementary material for: An online tool for mapping insecticide resistance in major Anopheles vectors of human malaria parasites and review of resistance status for the Afrotropical region
Source: Parasit Vectors. 2014 Feb 21;7:76. doi: 10.1186/1756-3305-7-76 (PMC3942210; doi:10.1186/1756-3305-7-76)
Supplement: Additional file 7 — Number of countries in Africa for which resistance mechanisms were detected in at least one population of Anopheles spp. Testing for resistance mechanisms in An. funestus s.s. has been sparse. [file 1756-3305-7-76-S7.pdf]

Number of countries in Africa for which resistance mechanisms were detected in at least one population of *Anopheles spp.*[26][§].

	**Time period^**	**2000 and prior**		**2001 - 2003**		**2004 - 2006**		**2007 - 2009**		**2010 - 2012**	
**Species or complex**	**Insecticide class**	*D*	*ND*	*D*	*ND*	*D*	*ND*	*D*	*ND*	*D*	*ND*
An. gambiae s.l.*	Oxidases	2	0	1	0	6	0	8	1	3	1
	Esterases	2	1	1	0	4	1	5	2	4	0
	GSTs	3	1	1	0	6	0	5	7	3	1
	kdr (L1014S)	4	2	5	6	8	8	8	8	3	7
	kdr (L1014F)	0	1	10	4	16	3	16	5	9	4
	Ace-1R	1	0	0	0	3	2	3	7	3	1
An. gambiae s.s.	Oxidases	1	0	0	0	4	0	2	0	3	0
	Esterases	1	0	0	0	1	1	2	0	3	0
	GSTs	1	1	0	0	3	0	2	0	2	1
	kdr (L1014S)	3	2	5	6	6	6	8	4	1	6
	kdr (L1014F)	4	1	10	3	12	2	14	3	8	2
	Ace-1R	0	0	0	0	3	0	3	3	3	1
An. arabiensis	Oxidases	1	0	1	0	3	0	3	1	0	2
	Esterases	1	0	1	0	1	0	2	2	0	2
	GSTs	1	0	1	0	3	0	1	1	0	2
	kdr (L1014S)	0	0	2	0	3	4	2	7	2	3
	kdr (L1014F)	0	1	1	4	6	4	4	5	4	3
	Ace-1R	0	0	0	0	0	3	1	4	0	1
An. funestus s.l.*	Oxidases	1	0	1	0	2	0	4	0	0	0
	Esterases	1	0	1	0	2	0	3	1	0	0
	GSTs	1	0	1	0	2	0	4	0	0	0
	kdr (L1014S)	0	0	0	0	0	0	0	3	0	1
	kdr (L1014F)	0	0	0	0	0	1	0	3	0	1
	Ace-1R	0	0	0	0	0	0	0	3	0	0

[§] Data sources and extraction, compilation and verification processes are outlined in Methods section
[#] Includes all species within the complex plus non-differentiated species
^ Refers to year of commencement of field collections
D = number of countries for which the specified mechanism was detected in at least one population of the species/complex [26]. For target site mechanisms this refers to detection of the specified alleles; for metabolic mechanisms this refers to field populations exhibiting significantly higher enzyme activity and/or gene expression levels than that of the reference susceptible strain (using biochemical assays and/or detox chip microarrays).
ND = number of countries for which the specified mechanism was tested for but was not detected [26]
